# Data for semi-permanent cationic coating for protein separations

**DOI:** 10.1016/j.dib.2020.105123

**Published:** 2020-01-11

**Authors:** C.L. Crihfield, C.J. Kristoff, L.M. Veltri, C.A. Wilson, W.M. Penny, L.A. Holland

**Affiliations:** C. Eugene Bennett Department of Chemistry, West Virginia University, Morgantown, WV, 26506, USA

**Keywords:** Capillary electrophoresis, Semi-permanent coating, Surface modification, Protein adsorption, pH-stability, Electroosmotic flow

## Abstract

Protein separations and analyses are fundamental to fields of study that include biochemistry, biology, physiology, drug discovery, pharmaceuticals, as well as agricultural and food based industries. Here, we provide the data from a novel phospholipid-cetyltrimethylammonium bromide coating capable of separating cationic and anionic proteins with high efficiency. Capillary electrophoresis separations of protein standards were utilized to characterize the performance of the novel coating. Using capillary electrophoresis with UV absorbance detection a working pH range of 4–9 was identified, with reproducibility in time ≤1% relative standard deviation, and plate counts for proteins as high as 480,000 plates (lysozyme, pH 7). Further details and results from these data are available in the work reported by Crihfield et al. and can be accessed at https://doi.org/10.1016/j.chroma.2019.460397 [1].

**Table:** Specifications Table

Subject	Analytical Chemistry
Specific subject area	Protein Separations, Capillary Electrophoresis, Bioanalysis
Type of data	Table Image Graph Figure
How data were acquired	Separations were performed using a P/ACE MDQ or MDQ Plus (Sciex, Redwood City, CA) with UV–vis absorbance detection at 200 nm.
Data format	Raw Analyzed
Parameters for data collection	Proteins were purchased and used as standards. A human serum standard was purchased and depleted of albumin and immunoglobulins with a kit.
Description of data collection	Data collection and analyses were performed using 32 Karat Software versions 7.0 or 10.2.
Data source location	Morgantown, West Virginia, United States of America
Data accessibility	Raw data are submitted to Mendeley https://data.mendeley.com/datasets/68ytr8mcrg/2
Related research article	C.L. Crihfield, C.J. Kristoff, L.M. Veltri, W.M. Penny, L.A. Holland, Semi-permanent Cationic Coating for Protein Separations, Journal of Chromatography, A, **2019**. 1607(6): p. 460397 https://doi.org/10.1016/j.chroma.2019.460397

**Table:** 

**Value of the Data** • A coating is described that provides efficient separations of anionic and cationic proteins simultaneously. • Scientists separating proteins for analysis or identification can benefit from this research. • The coating developed here can be utilized for improved protein separations applicable to biological research. • Protein assays could be operated at pH values ranging from 4 to 9 with stable electroosmotic flow

## 1 Data

**Fig. 1:**
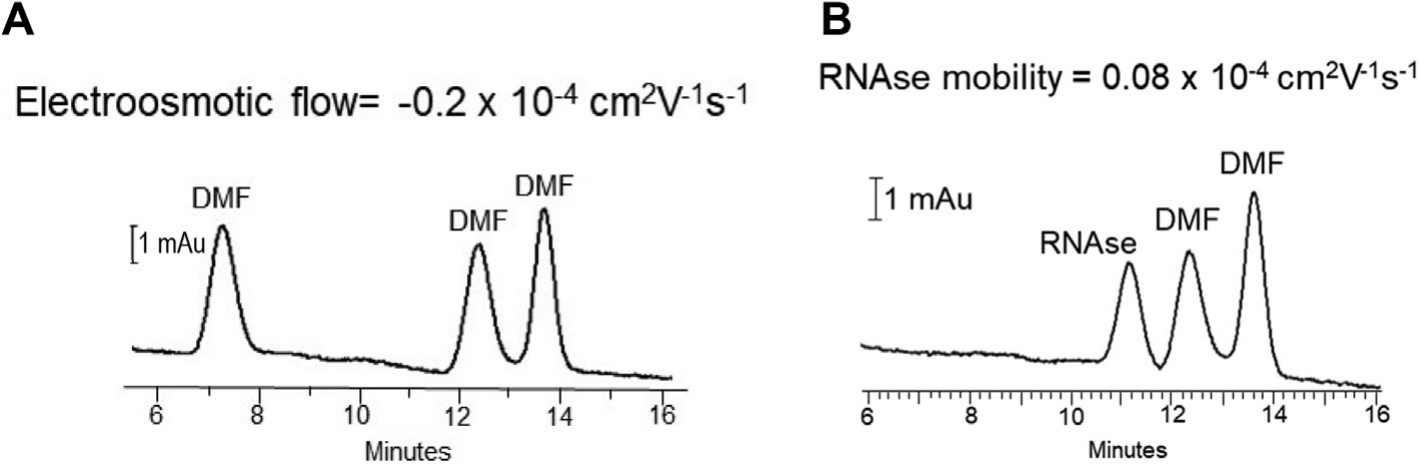
**A**. The electroosmotic flow in a phospholipid coated capillary was determined to be −0.2 × 10^−4^ cm^2^V^−1^s^−1^ using the method previously reported in the literature [2]. For this analysis, the specified pressure and push step into the capillary was 3.4 kPa (0.5 psi) 5 min and the electrophoresis time was 3 min at 24 kV. In **B**, the electrophoretic mobility of Ribonuclease A (RNAse) in a phospholipid coated capillary was determined with slight modification and required only two injections, the first one containing DMF and ribonuclease A and the second containing only DMF. The push step was 3.4 kPa (0.5 psi) for 5 min and the electrophoresis time was 3 min at 24 kV.

**Fig. 2:**
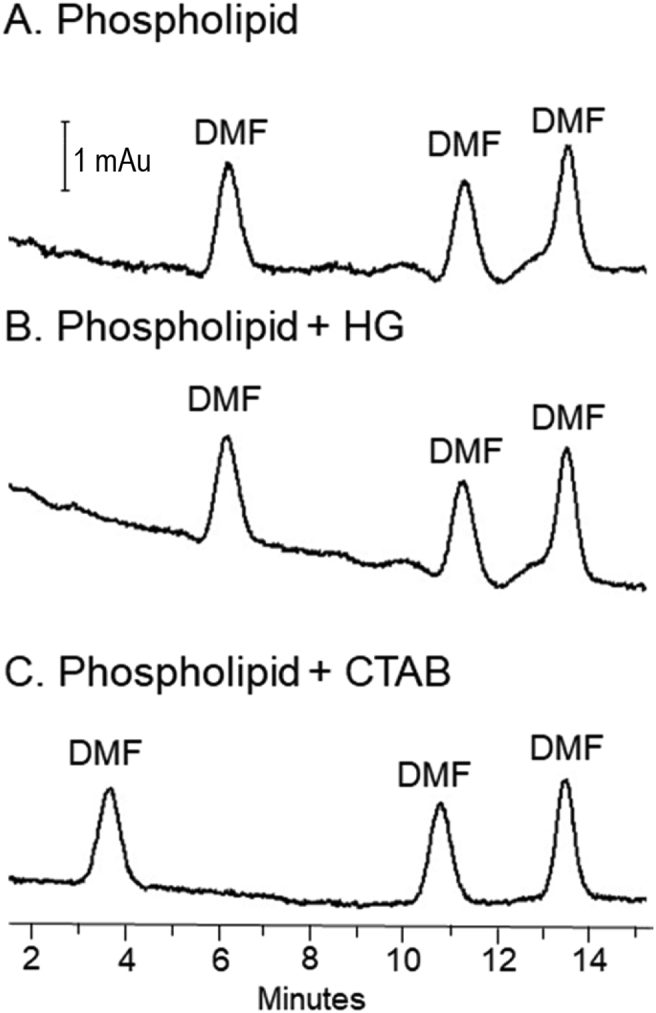
The electroosmotic flow for a phospholipid coated capillary (A) for phospholipid capillary coated with the tetramethyl ammonium bromide head group (HG) of CTAB (B) and for phospholipid capillary coated with CTAB (C) was determined using the method described in Fig. 1, with some modifications. The phospholipid (A) and phospholipid with HG (B) were analyzed with no modification, which resulted in electroosmotic flow of −0.2 × 10^−4^ cm^2^V^−1^s^−1^. Due to the high electroosmotic flow in the phospholipid with CTAB capillary (C), the method was modified to have a 3.4 kPa (0.5 psi) 7 min push step and 30 s electrophoresis to keep the peaks between the injection and detection sites. The phospholipid with CTAB capillary had an electroosmotic flow of −4.2 × 10^−4^ cm^2^V^−1^s^−1^.

**Fig. 3:**
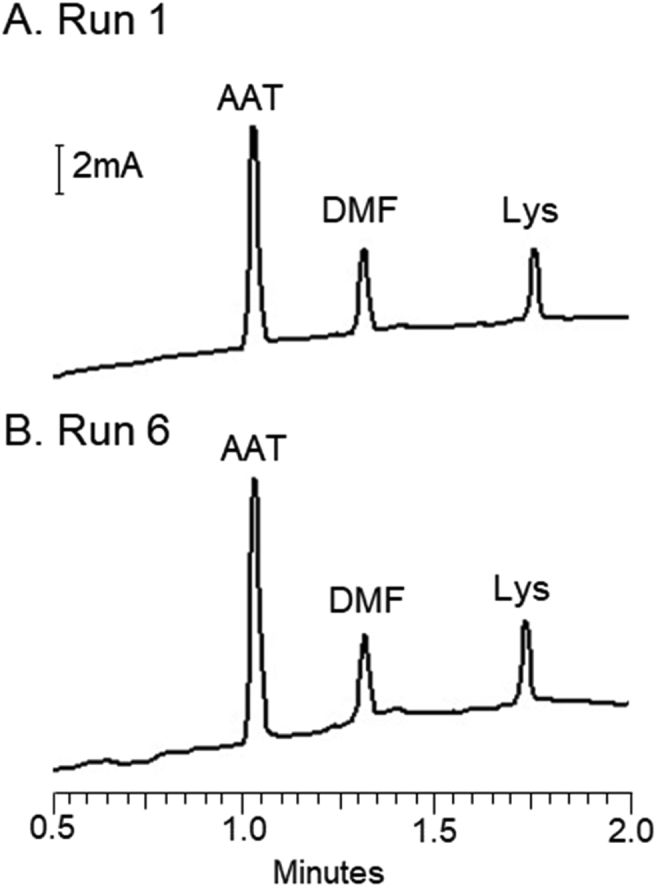
The absence of protein adsorption when the phospholipid-CTAB coating is applied was proven by performing consecutive analyses of α-1-antitrypsin, neutral marker (DMF), and lysozyme without flushing between each run. The migration times of the proteins and neutral marker vary by 1% from run 1 (A) to run 6 (B), which indicates that no adsorption occurred.

**Fig. 4:**
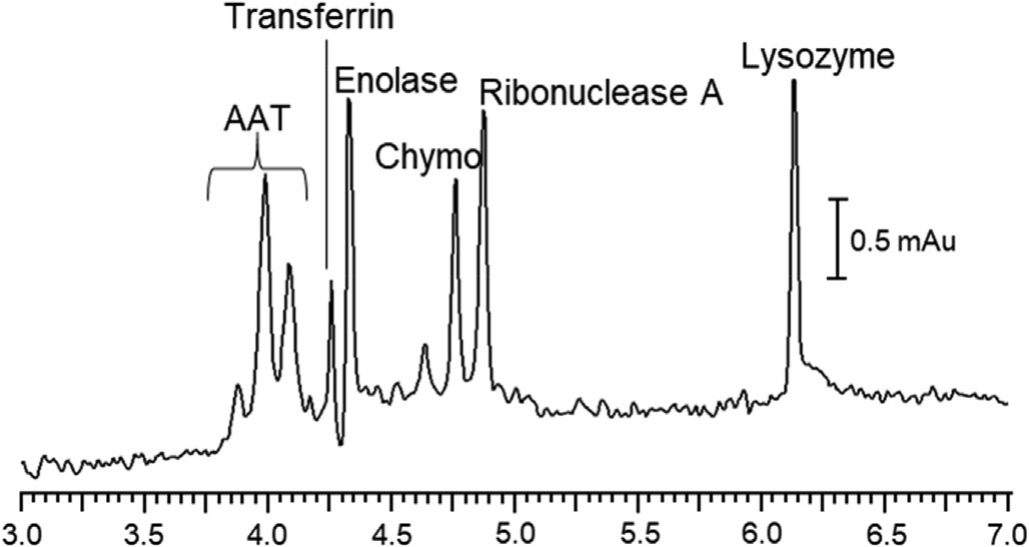
An electropherogram of a 6 protein mixture obtained using a phospholipid-CTAB coated fused silica capillary. The background electrolyte is ammonium acetate (pH of 6.5). Run conditions: UV detection at 200 nm; L_tot_ = 60 cm; L_eff_ = 50 cm; 24 kV applied voltage in reversed polarity. Standards are injected at 3.4 kPa (0.5 psi) for 5 s.

The information presented is a summary of data for the electroosmotic flow (Fig. 1A) and the electrophoretic mobility measurement for ribonuclease A (Fig. 1B) in a phospholipid coated capillary, and a comparison of the separation efficiency achieved using bare fused silica and phospholipid coated capillaries (Table 1). Information characterizing the phospholipid-cetyltrimethylammonium bromide (CTAB) coating is also included, such as the electroosmotic flow measurements verifying the role of the hydrophobic tail insertion (Fig. 2) and impact of concentration (Table 2), flush time (Table 3), and pH (Table 4) on electroosmotic flow. A comparison of the efficiency between the phospholipid and phospholipid-CTAB coated capillaries (Table 5) and evidence that protein does not accumulate on the surface over 6 runs (Fig. 3) is provided, as well as a comparison of the plates/meter for phospholipid-CTAB separations with an effective length of 10 cm versus 50 cm (Table 6). The impact of surface modification on the protein electrophoretic mobilities (Table 7) and the influence of pH on migration time (Table 8) and plate count (Table 9) are presented. Compatibility with a background electrolyte composed of ammonium acetate is demonstrated with an image of an electropherogram (Fig. 4).

**Table 1:** Impact of capillary surface on efficiency.

	Average plates (RSD)^a^				
	AAT^b^	Tf	Enol	RNAse	Lys
Bare fused	N/A	5000 (21)	14,000 (18)	11,000 (9)	N/A^c^
Phospholipid	N/A	11,000 (2)	79,000 (4)	N/A^d^	16,000 (11)

a Separations obtained using a background electrolyte of 50 mM sodium phosphate 50 mM sodium acetate buffered at pH 7, a 25 μm internal diameter fused silica capillary with a total length of 60 cm, an effective length of 10 cm and E = 400 V/cm. Relative standard deviation (RSD) and average are based on *n* = 6 measurements.

b α-1-antitrypsin not included because the isoforms produced multiple peaks.

c Adsorption of lysozyme to the silica wall, prevented calculation of plate count.

d Ribonuclease A migrated too slowly to be seen within the separation timeframe.

**Table 2:** Impact of CTAB concentration on electroosmotic flow.

[CTAB], μM	Electroosmotic flow x 10^−4^ cm^2^V^−1^s^−1^ (RSD)
50	2.04 (3)
100^a^^,^^b^	3.14 (0.3)
300^b^	3.14 (0)
500^b^	3.14 (0.2)

a Separation conditions as described in Table 1. Relative standard deviation (RSD) and average at 100 μM CTAB were obtained from *n* = 3 measurements, while all other concentrations were obtained from *n* = 5 measurements.

b These concentrations are statistically the same as determined with ANOVA (*ρ* < 0.2516, F value 1.752 < 5.14).

**Table 3:** Impact of buffer flush time on electroosmotic flow.

Flush time (min)^a^	Electroosmotic flow x 10^−4^ cm^2^V^−1^s^−1^ (RSD)^b^
4.5	3.10 (0)
7.5	3.10 (0.3)
10	3.14 (0)
15	3.11 (0.3)
30	3.11 (0.2)
60	3.12 (0.6)
120	3.15 (0.2)

a The flush time listed is the buffer flush after the CTAB flush and programmed wait step to prevent contamination in the CTAB flushing sequence.

b Separation conditions as described in Table 1. Relative standard deviation (RSD) and average were obtained with *n* = 3 measurements.

**Table 4:** Impact of pH on electroosmotic flow.^a^^,^^b^

[CTAB] (μM)	pH	Electroosmotic flow x 10^−4^ cm^2^V^−1^s^−1^ (RSD)
100	9	3.13 (0.9)
	8	3.05 (1)
	7	3.16 (0.6)
	6	3.62 (0.4)
	5	3.78 (0.4)
	4	3.78 (0.5)

a Relative standard deviation (RSD) and average were obtained with *n* = 10 measurements.

b Separation conditions were as follows: L_tot_ = 60 cm, L_eff_ = 50 cm, 24 kV applied voltage in reverse polarity.

**Table 5:** Impact of capillary surface on efficiency.

	Average plates (RSD)^a^				
	AAT	Tf	Enol	RNAse	Lys
Phospholipid	N/A^b^	11,000 (2)	79,000 (4)	N/A^c^	16,000 (11)
Phospholipid/CTAB	N/A^b^	12,000 (0.4)	16,000 (4)	20,000 (0.3)	32,000 (4)

a Separation conditions as described in Table 1. Relative standard deviation (RSD) and average were obtained are based on *n* = 6 measurements.

b AAT theoretical plate counts were excluded due to presence of multiple isoforms, making plate count measurements not applicable (N/A).

c Ribonuclease A was not observed in the time frame of the phospholipid analysis because of the low mobility.

**Table 6:** Impact of capillary length on plates/meter.^a^

	Average plates/meter (RSD)				
	AAT^b^	Tf	Enol	RNAse	Lys
50 cm L_eff_	N/A	170,000 (20)	730,000 (3)	730,000 (3)	1,000,000 (3)
10 cm L_eff_	N/A	110,000 (10)	130,000 (20)	190,000 (20)	250,000 (20)

a Effective length is specified. Other separation conditions as described in Table 1. Relative standard deviation (RSD) and average were obtained are based on *n* = 6 measurements.

b AAT was excluded due to the presence of multiple isoforms, making plate count measurements not applicable (N/A).

**Table 7:** Impact of capillary surface on mobility.

	Electrophoretic Mobility x 10^−4^ cm^2^V^−1^s^−1^ (RSD)^a^				
	AAT	Tf	Enol	RNAse	Lys
Phospholipid	−0.91 (4)^b^	−0.52 (6)	−0.26 (9)	0.08 (10)^c^	0.74 (6)
CTAB/Phospholipid	−0.83 (0.9)	−0.48 (2)	−0.25 (2)	0.07 (4)	0.75 (0.5)

a Separation conditions as described in Table 1. Relative standard deviation (RSD) and average are based on *n* = 6 measurements except where noted.

b For clarity, only the mobility of the largest AAT peak is reported in this table.

c Electrophoretic mobility was determined as described in Fig. 1.

**Table 8:** Effect of pH on migration time and precision.

	Average Migration Time, min (%RSD)^a^					
	pH 9^b^	pH 8	pH 7	pH 6	pH 5	pH 4
α-1-Antitrypsin^c^	4.83 (0.4)	5.03 (1)	5.10 (0.4)	4.74 (0.4) 4.82 (0.4)	4.88 (0.3) 4.91 (0.3) 5.05 (0.4)	N/A^d^
Transferrin	5.28 (1)	5.65 (1)	5.65 (0.5)	5.29 (0.4)	5.48 (0.2)^e^	N/A^d^
Enolase	5.43 (1)	6.03 (2)	6.08 (0.5)	5.74 (0.7)^f^	5.96 (0.5)	7.45 (2)
α-Chymo-trypsinogen A	5.94 (4)	6.71 (2)	6.72 (0.4)	6.40 (0.6)	6.54 (0.5)	7.17 (1)
Ribonuclease A	6.18 (1)	6.77 (2)^g^	6.83 (0.4)	6.58 (0.7)	6.80 (0.5)	7.66 (1)
Lysozyme	7.74 (3)	8.73 (3)	8.78 (0.6)	8.50 (0.9)	8.48 (0.7)	9.17 (2)

a Relative standard deviation (RSD) and average are based on *n* = 20 measurements for the proteins and *n* = 10 measurements for dimethylformamide, except as noted below.

b Measurements obtained at pH 9 are based on *n* = 10.

c α-1-antitrypsin isoforms are resolved at pH 6 and 5, which resulted in multiple migration times.

d α-1-antitrypsin and transferrin were excluded from the sample at pH 4.

e Transferrin was excluded from the sample at pH 5 when dimethylformamide was included. (*n* = 10).

f Enolase was excluded from the sample at pH 6 when dimethylformamide was included. (*n* = 10).

g Ribonuclease A was excluded from the sample at pH 8 when dimethylformamide was included. (*n* = 10).

**Table 9:** Effect of pH on plate count.

	Average plates (RSD)^a^					
	pH 9	pH 8	pH 7	pH 6	pH 5	pH 4
α-1-Antitrypsin	100,000 (40)	90,000 (10)	64,000 (3)	47,000 (3)	350,000 (5)	N/A^b^
Transferrin	110,000 (9)	130,000 (10)	80,000 (3)	190,000 (7)	150,000 (12)^c^	N/A^b^
Enolase	180,000 (15)	250,000 (10)	360,000 (4)	270,000 (8)^d^	150,000 (30)	80,000 (40)^e^
α-Chymo-trypsinogen A	60,000 (20)^f^	330,000 (9)	340,000 (30)	380,000 (5)	300,000 (7)	230,000 (20)
Ribonuclease A	70,000 (40)	290,000 (7)^g^	400,000 (10)	400,000 (5)	310,000 (6)	230,000 (30)
Lysozyme	300,000 (10)	340,000 (30)	480,000 (7)	470,000 (5)	400,000 (6)	400,000 (10)

a Relative standard deviation (RSD) and average are based on *n* = 20 for the proteins and *n* = 10 measurements for the DMF, except as noted below.

b α-1-antitrypsin and transferrin were excluded from the sample at pH 4.

c Transferrin was removed at pH 5 when DMF was ran. (*n* = 10).

d Enolase was excluded from the sample at pH 6 when DMF was included. (*n* = 10).

e Enolase plate count could not be calculated in three of the analyses due to the baseline interference with where half height was integrated. (*n* = 17).

f Chymotrypsinogen A width could not be calculated when the peak migrated too closely to the ribonuclease A peak in three analyses. (*n* = 7).

g Ribonuclease A was excluded from the sample at pH 8 when DMF was included and when the peak migrated too closely to the α-chymotrypsinogen A peak for a width to be calculated in the last three analyses. (*n* = 7).

## 2 Experimental design and materials and methods

Capillaries were treated to contain a phospholipid-CTAB coating. Protein standards and/or dimethylformamide, which was used as a neutral marker, were separated with the coated capillaries. The performance metrics were determined through these experiments.

### 2.1 Capillary electrophoresis methods

The methods used were capillary electrophoresis and ultraviolet absorbance detection at 200 nm. Standard separations were performed using capillary electrophoresis. The proteins used in these studies were α-1-antitrypsin (AAT), transferrin (Tf), enolase (Enol), ribonuclease A (RNAse), lysozyme (Lys), and α-chymotrypsinogen A. Protein and dimethylformamide samples were prepared in 50 mM sodium phosphate acetate buffered at the same pH as the background electrolyte for the experiment except in the case of pH 4 experiments for which the protein samples were buffered at pH 7 to maintain stability. Samples were injected at 3.7 kPa (0.5 psi) for 5 seconds. In cases when electroosmotic flow was too slow for the use of a traditional capillary electrophoresis separation a fast and accurate method previously reported was used. For this method 3 sample injections are made in a single run with each injection segregated by background electrolyte. Voltage is applied to monitor the effect of electric field on the spacing between the analyte bands. In summary, the steps are to inject a plug of dimethylformamide, push it a specified distance in the capillary, inject a second plug of dimethylformamide, push it the same distance, apply voltage for a for a specified time, inject a third plug of dimethylformamide, and push all three plugs to the detection window. The magnitude of the electroosmotic flow is related to the difference between the distances of the first and second plug which are separated by pressure and the second and third plug which are separated by voltage. The method can be tuned to measure electroosmotic flow of different surfaces by either adjusting the electrophoresis time or the specified distance the plugs are pushed in, as long as the injected zones of the neutral marker are within the space between the detection window and the injection site.
